# Moya Moya Disease: A rare disease with a common presenting symptom in a chronic kidney disease patient

**DOI:** 10.12669/pjms.39.6.7953

**Published:** 2023

**Authors:** Sidra German, Sajid Islam Bhatti, Rubina Naqvi, Tajammul Waqar

**Affiliations:** 1 Sidra German, Department of Nephrology, Sindh Institute of Urology and Transplantation (SIUT), Karachi, Pakistan; 2 Sajid Islam Bhatti, Department of Nephrology, Sindh Institute of Urology and Transplantation (SIUT), Karachi, Pakistan; 3 Rubina Naqvi, Department of Nephrology, Sindh Institute of Urology and Transplantation (SIUT), Karachi, Pakistan; 4 Tajammul Waqar Department of Nephrology, Sindh Institute of Urology and Transplantation (SIUT), Karachi, Pakistan

**Keywords:** Moya Moya disease (MMD), Kidney replacement therapy (KRT), Chronic Kidney Disease (CKD)

## Abstract

Moya Moya Disease (MDD) is a rare cerebrovascular pathology. It is non atherosclerotic cerebrovascular disease characterized by bilateral internal carotid stenosis or occlusion, and abnormal vascular network at the base of the brain. Here we report a case of young female who presented in emergency with complaints of jerky movements of limbs for six months and history of recently developed unusual high blood pressure which was followed by uremic symptoms. Her workup revealed severe renal dysfunction required kidney replacement therapy (KRT) i.e., hemodialysis. During hospital stay her mental status deteriorated with a drop in GCS. Brain imaging performed and she found to have MMD. Her clinical course continued to deteriorate despite of extensive work up and aggressive management, she died eventually.

## INTRODUCTION

Moya Moya Disease (MMD) is a rare, chronic and progressive cerebrovascular disease, characterized by stenosis of bilateral internal carotid arteries with abnormal network at the base of brain.^1^ Extra cranial vessels mainly involve renal artery.^2^ It is rare disease with reported incidence of 0.086 per 100,000 population^3^, more common in East Asian countries like, Japan, Korea and China with slight female predominance (2:1). It has bimodal age of onset, with children presenting around age of five years and adult presenting around age of 40. Its presentation varies in adult, it is typically a hemorrhage, while in children it is stroke or transient ischemic attack and may present with headache and seizure in both of the population.^4^ It usually affects bilateral internal carotid arteries, however unilateral presentation of underlying pathology is known as Moya Moya syndrome and is associated with conditions like sickle cell disease, neurofibromatosis Type-1, and Down syndrome.^4^ We present here a case of this rare disease occurring in a young woman.

## CASE PRESENTATION

A 23 years old female, resident of Karachi, unmarried, with no known prior comorbids, presented to us in emergency room with complaints of easy fatigability, poor appetite, nausea and decreased urine output of two weeks duration. She also mentioned jerky movement of all four limbs for the last six months and Hypertension for last four months for which she was prescribed amlodipine 5mg once daily four months back. Examination on arrival revealed BP: 180/110, Pulse 80/min, Temperature 98°F and respiratory rate 20 breaths/min. She was anemic but there was no jaundice, cyanosed, clubbing, koilonychias or edema feet. CNS, motor and sensory examination was normal with intact higher mental functions and cranial nerves. Glasgow Coma Scale (GCS) was 15/15. Chest examination revealed bilateral crepitations on auscultation and a gallop sound at precordial area. Abdomen was soft and non-tender with no visceral enlargement. Laboratory parameters revealed grossly abnormal kidney functions with serum creatinine of 13.5 mg/dl, urea 191mg/dl, Na 133mEq/L, K 4.1mEq/L, Cl 91mEq/L and HCO3 15mEq/L, Hemoglobin was 7.2g/dL, TLC 10.2 x10^9^/L, Platelet 424 x 10^9^/L. On ultrasonography kidney were measured as 7cm each, with increased echogenicity. Chest roentgenogram revealed signs of pulmonary edema. She was commenced on hemodialysis with temporary dialysis catheter.

**Fig.1:**
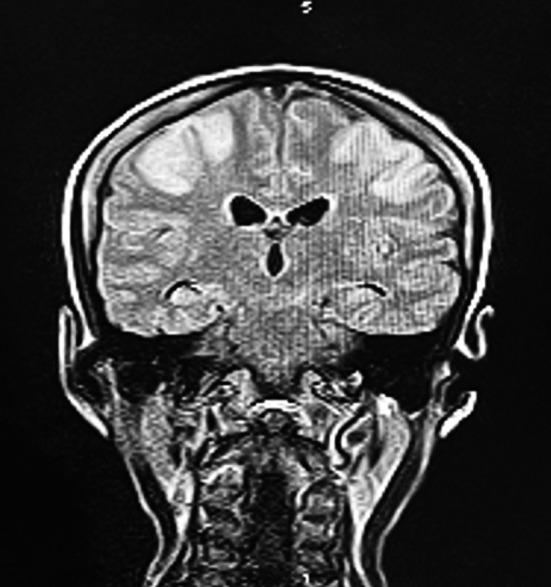
MRI brain showing hyperintense signal in bilateral parietal region. (Picture courtesy Dr. Muhammad Farid, dept. of Radiology, SIUT).

**Fig.2:**
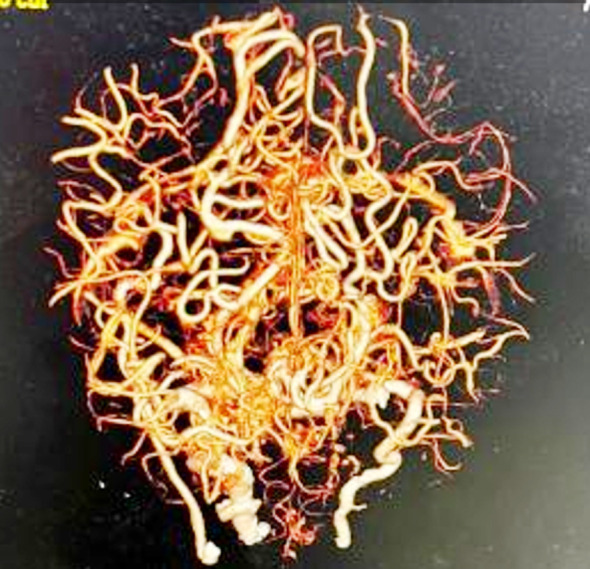
CT angiogram of brain showing coiled network of vessels at the base of brain giving puff of smoke appearance “Moya Moya”. (Picture courtesy: Dr. M. Farid, Dept. of Radiology, SIUT)

During her hospital stay she developed generalized tonic clonic seizures (GTCs) multiple times and dropped her conscious level, GCS declined to 7/15. She was electively intubated and kept on mechanical ventilation. During workup for the cause of GTCs her metabolic profile found normal and cerebrospinal fluid analysis was inconclusive. Brain imaging performed, CT scan brain done, which raised the suspicion of bilateral parietal regions infarct, this was confirmed with MRI brain, which showed bilateral parietal lobe infarct as evidenced by hyper intense signals in T1 images (Fig.1). CT angiogram of brain revealed non- visualization of bilateral middle cerebral, anterior cerebral arteries and Circle of Willis. Numerous collaterals from posterior carotid and extra carotid circulation supplying anterior portion of brain were found and these findings are suggestive of Moya Moya disease (Fig.2). Unfortunately, CT angiogram of abdomen could not have done to rule out renal artery stenosis as patient did not survive.

## DISCUSSION

MMD is a chronic, progressive, cerebrovascular disease characterized by occlusion of bilateral internal carotid arteries at their distal portions.^4^ As a result, collateral network of leptomeningeal vessel formed at the base of brain giving appearance of ‘puff of smoke’ known as Moya Moya in Japanese.^5^ There are four defined types of MMD, ischemic, hemorrhagic, epileptic and other. It has unique clinical features with two peaks of age distribution at age of five years and at 40. Our patient was different in a way that she was not at any of this peak. The cause of chronic kidney disease remained unclear from history in our patient, but we as a routine see many young patients presenting in our emergency room with advanced uremia and small kidneys indicating a long term pathology going on.

Pediatric population usually have ischemic attack where as adults have both hemorrhagic and ischemic.^6^ It is more common in females as compared to males and has highest incidence in Japan.^4^ MMD has some genetic association linked to chromosome 17 in Japanese as well as with other demographics.^5^

It can present with recurrent TIA, epilepsy, stroke (hemorrhagic or infarct), hemiparesis, dysarthria, aphasia, and cognitive impairment.^7^ Reno vascular hypertension is considered to be one of the important causes of hypertension in patients with MMD.^8^ It is diagnosed by catheter angiography or magnetic resonance angiography showing stenosis of the internal carotid artery, anterior cerebral artery, or middle cerebral artery.^7^ Surgical revascularization is the mainstay of treatment. It is done via direct, indirect and combined approaches. Direct include extracranial-intracranial (EC-IC) arterial bypass surgery. It is commonly used in adult patients. The indirect encephalo duro arterio syangiosis (EDAS) procedure involves laying down a branch of the superficial temporal artery onto the brain surface, allowing for neovascularization to supply blood flow to the tissue indirectly rather than a direct anastomosis. The EDAS procedure is more commonly performed in children.^4^ Medical management include aspirin and clopidogrel, lifelong anticoagulation with apixaban followed by seizure prophylaxis, antihypertensive medications, cholesterol management with statins and dietary modification for healthy lifestyle.^7^ We took neurosurgery on board but because of the status of patient, they advised to manage the case conservatively. She was on combination of antihypertensive and antiepileptic drugs.

## CONCLUSION

Rare disease like MMD can be missed from diagnosis if not considered in differential. Our case brings forth the importance of considering MMD in the differential diagnosis for patients suffering from repeated jerky movements/GTCs even in younger age.

### Authors ‘contribution:

**SG**: Identified the case thought about presenting it and prepared baseline manuscript. She is also responsible for the accuracy of the work.

**SB**: Guided through sequencing the diagnostics and literature search.

**RN:** Significantly revised the manuscript.

**TW**: Assisted through patient going under all investigations, follow-ups and literature search.
